# Serial image analysis of *Mycobacterium tuberculosis* colony growth reveals a persistent subpopulation in sputum during treatment of pulmonary TB

**DOI:** 10.1016/j.tube.2016.03.001

**Published:** 2016-05

**Authors:** David A. Barr, Mercy Kamdolozi, Yo Nishihara, Victor Ndhlovu, Margaret Khonga, Geraint R. Davies, Derek J. Sloan

**Affiliations:** a Wellcome Trust Liverpool Glasgow Centre for Global Health Research, University of Liverpool, 70 Pembroke Place, Liverpool, L69 3GF, UK; b Brownlee Centre for Infectious Disease, Gartnavel General Hospital, 1053 Great Western Road, Glasgow, G12 0YN, UK; c Department of Microbiology, College of Medicine, University of Malawi, Mahatma Gandhi Road, Blantyre 3, Malawi; d Queen Elizabeth Central Hospital, P.O. Box 95, Blantyre 3, Malawi; e Institute of Infection and Global Health, The Ronald Ross Building, University of Liverpool, 8 West Derby Street, Liverpool, L69 7BE, UK; f Malawi Liverpool Wellcome Trust Clinical Research Programme, College of Medicine, University of Malawi, Mahatma Gandhi Road, Blantyre 3, Malawi; g Liverpool Heart and Chest Hospital, Thomas Drive, Liverpool, L14 3PE, UK; h Liverpool School of Tropical Medicine, Pembroke Place, Liverpool, L3 5QA, UK

**Keywords:** Pharmacodynamics, Biomarkers, Persisters, Drug tolerance, TTD, time to detection in MGIT, CFU, colony forming units, RGR, radial growth rate

## Abstract

Faster elimination of drug tolerant ‘persister’ bacteria may shorten treatment of tuberculosis (TB) but no method exists to quantify persisters in clinical samples. We used automated image analysis to assess whether studying growth characteristics of individual *Mycobacterium tuberculosis* colonies from sputum on solid media during early TB treatment facilitates ‘persister’ phenotyping. As Time to Detection (TTD) in liquid culture inversely correlates with total bacterial load we also evaluated the relationship between individual colony growth parameters and TTD. Sputum from TB patients in Malawi was prepared for solid and liquid culture after 0, 2 and 4 weeks of treatment. Serial photography of agar plates was used to measure time to appearance (lag time) and radial growth rate for each colony. Mixed-effects modelling was used to analyse changing growth characteristics from serial samples. 20 patients had colony measurements recorded at ≥1 time-point. Overall lag time increased by 6.5 days between baseline and two weeks (p = 0.0001). Total colony count/ml showed typical biphasic elimination, but long lag time colonies (>20days) had slower, monophasic decline. TTD was associated with minimum lag time (time to appearance of first colony1). Slower elimination of long lag time colonies suggests that these may represent a persister subpopulation of bacilli.

## 1 Introduction

To meet global tuberculosis (TB) control targets, shorter duration treatment regimens are needed [1]. Recent attempts to shorten therapy for drug susceptible TB to less than 6 months in phase III clinical trials have resulted in high rates of treatment failure and relapse [2], [3], [4]. Such unfavourable outcomes are often attributed to the capacity of phenotypically drug-tolerant ‘persister’ bacilli to survive antibiotic exposure [5], [6]. Development of techniques to monitor the specific activity of novel antimicrobial regimens against persister cells would significantly advance TB therapeutics research, and help select drug combinations with greater treatment shortening potential [7].

Bacilli in sputum have traditionally been quantified by counting colony forming units (CFUs) after incubation on solid media. Mathematical modelling of serial CFU counts during TB treatment has shown biphasic bacterial killing, supporting the hypothesis that different sub-populations of *Mycobacterium tuberculosis* are eliminated at different rates. However, total CFU counts do not selectively identify persister organisms. *In vitro* experiments have used automated image analysis for detailed study of individual *Escherichia coli* colonies, measuring their time to appearance (lag-time) and radial growth rate (RGR) on culture plates under a range of conditions [8]. In these models, the primary adaptation to antibiotic stress was development of drug tolerance through a population shift in CFU lag-time distribution, without generation of resistance mutations [9]. Incorporation of similar methodology to sputum colony counting studies is required to test whether differential lag-time or RGR measurements identify a persister TB phenotype from clinical samples.

Time to detection (TTD) in liquid culture is an alternative means of *M tuberculosis* quantification [10]. There is a known negative correlation between TTD and CFU counts, and recent studies have modelled treatment response on the basis of changes in TTD over time on therapy [11]. Whilst liquid culture offers several advantages, including more successful revival of pesisters than solid media, the precise relationship between TTD and CFU counts is incompletely understood and requires further study [12], [13].

For the current study we adapted the *in vitro* colony imaging approach to provide lag-time and RGR measurements from individual *M tuberculosis* CFUs recovered from serial sputum samples of adult pulmonary TB patients. We assessed whether heterogeneity in lag-time and RGR measurements revealed a sup-population of persister organisms. We also compared these parameters to TTD results from liquid culture of the same specimens, to provide new information on the relationship between individual CFU growth and TTD of the overall sample.

## 2 Methods

### 2.1 Patient selection and sample collection

The study was conducted in Blantyre, Malawi as part of a large multicentre prospective cohort study named PanACEA Biomarkers Expansion Project (PanBioME). Patients aged 18–65 years with smear positive pulmonary TB (grade 2–3+ by IUATLD criteria on Ziehl-Neelsen staining [14]) were recruited to PanBioME. Exclusion criteria included previous TB treatment in the last 5 years, or inability to complete the first two weeks of first-line treatment. Sputum was obtained by combining one overnight collection at the patient's home with an assisted early morning collection at the Queen Elizabeth Central Teaching Hospital tuberculosis service, 0, 14 and 28 days into therapy. Ethical approval was granted by the Research Ethics Committee, College of Medicine, University of Malawi.

### 2.2 Sample preparation and quantitative bacteriology

Highly viscous sputum was liquefied by glass-bead vortexing with a volume of distilled water <15% of the sample volume. Two–four ml of un-decontaminated samples were then homogenised by addition of an equal volume of working strength dithiothreitol (Oxoid, SR0023A) and repeated light vortexing over the next 30 min. Ninety millimetre diameter plates were prepared with selective media (Middlebrook 7H11 oleic acid albumin agar with MAST Selectatabs™, and carbedazim, according to a previously published protocol from our laboratory [15]) to a depth of ∼4 mm.

Six serial ten-fold dilutions (neat to 10^−6^) of the processed sputum were prepared using the micropipetting method and phosphate buffered saline (PBS) diluent, with vortexing and micropipette tip change between each dilution step [15]. Each dilution was inoculated in duplicate, using a disposable loop, onto selective plates. Plates were then sealed using 12 mm Micropore™ tape, stored inside airtight polyethylene specimen bags and storage boxes and incubated at 37 °C in darkness, media-surface upside with tri-weekly shuffling. Colony counts were performed at 4 weeks, and used to calculate CFU/ml counts for original sputum by back-calculation accounting for inoculum volume and dilution factor. A minimum of one colony per specimen was identified as containing acid-fast bacilli on Ziehl-Neelson microscopy, including any colonies regarded as having ambiguous morphology.

In parallel, aliquots of each sample was processed for liquid culture in BACTEC™ MGIT™ 960 Mycobacterial detection system (Becton Dickinson). One millilitre homogenised sputum was decontaminated with 1 ml N-acetyl-l-cysteine/NaOH 3% for 15 min before inoculation into BBL-MGIT™ 7 ml tubes prepared as per manufacturer instructions – 7 ml Middlebrook 7H9 broth base supplemented with oleic acid, albumin, dextrose, and catalase (BBL-MGIT™ OADC) and polymixin B, amphotericin B, nalidixic acid, trimethoprim (BBL-MGIT™ PANTA). Isolates which signalled positive were confirmed as *M. tuberculosis* if they demonstrated acid-fast bacilli with cording and MPT64Ag positivity on commercial identification kits (Becton Dickinson). TTD in hours was retrieved from BACTEC™ MGIT™ 960 software. Only TTD results from confirmed *M. tuberculosis* positive cultures were used in the analysis. Specimens which had not signalled positive by 8 weeks were reported as negative.

### 2.3 Digital imaging to assess colony lag time and RGR

Media-side down plates were back-illuminated in a custom built light box by a low-heat LED panel light for photographing three times per week. A compact digital camera (Nikon™ Coolpix™ S6700 2.1 megapixel, settings: “MACRO”, “high quality, large image”) was fixed to the light box 20 cm from plate position. Plate edges were marked to allow standardised positioning in serial photographs and additional markings, visible in final images, permitted a post-hoc check for standardised positioning relative to the camera (Figure S1, available as online supplement).

The size and position of colonies in each digital image were measured using open source software (OpenCFU v3.9.0 beta) [16]. Combined colony observations from serial images of the same plate were used to extract growth curves for each unique colony.

Several colony growth models (including all those previously described in the literature [17], [18]) were assessed for fit against observed growth data. A simple linear model of colony radius as a function of time for initial colony growth (i.e. after censoring plateau phase observations) showed better fit than a monoexponential, biexponential, or power-law function. Therefore, an individual linear model was fitted to the initial growth of each colony using the ordinary least squares method. Colony RGR was defined as the slope co-efficient, and lag time as the x intercept, of this model (Figure S2, available as online supplement). ‘Minimum lag time’ was defined as the shortest colony lag time observed amongst colonies grown from a given sample.

Although every plate, at every dilution of each clinical sample was imaged, prior work on soil microbes [19], and optimization experiments on *M tuberculosis* laboratory strain H37Rv (Figure S3, available as online supplement) have shown that the concentration of bacteria inoculated onto a culture plate (plating density) influences the probability a CFU will form a colony, and its lag time in doing so. Therefore, analysis of individual colony growth kinetics from clinical samples in this study was restricted to the plate dilutions of each specimen which yielded a final count of 1–50 colonies per plate.

### 2.4 H37Rv control plates

Two-hundred and sixty-nine *M tuberculosis* colonies grown from H37Rv laboratory strain were analysed in the same way as the clinical isolate colony growth for comparison.

### 2.5 Statistical analysis

The data structure for this study was hierarchical and nested, with serial measures of colonies, within plates, from repeated sampling of the same individuals at different time points. Therefore, statistical methods that accounted for non-independence of the observations (or their error terms) were employed, including use of summary measures and mixed-effects modelling [20], [21]. In particular, linear mixed effects models were used to assess changes in colony growth kinetics from patient samples collected at different study visits. Correlations of TTD with solid media growth characteristics were assessed with R^2^ from linear regression. All data management and statistical analysis was conducted in R Studio (Version 0.98.1102) [22].

## 3 Results

### 3.1 Patients, samples and quantitative bacteriology

Twenty-one patients were recruited; 20 patients had colony growth at one or more treatment timepoints. Positive samples were required for analysis of colony growth kinetics. 35 sputum specimens fulfilled these criteria; their time-points of collection, CFU counts and TTD data are summarised in Table 1.

### 3.2 Lag time and RGR of colonies recovered from clinical isolates vary over time

Approximately 16000 digital images of inoculated plates were captured across all specimens at all timepoints. However, based on criteria that only colonies from plate dilutions which yielded a final colony count of 1–50 CFU, images of 1352 colonies were analysed, with a median of 6 measurements of each colony.

There was no correlation between lag time and RGR for individual colonies (Pearson's r = −0.03), suggesting that these measurements are independent measures of colony behaviour.

**Figure 1:**
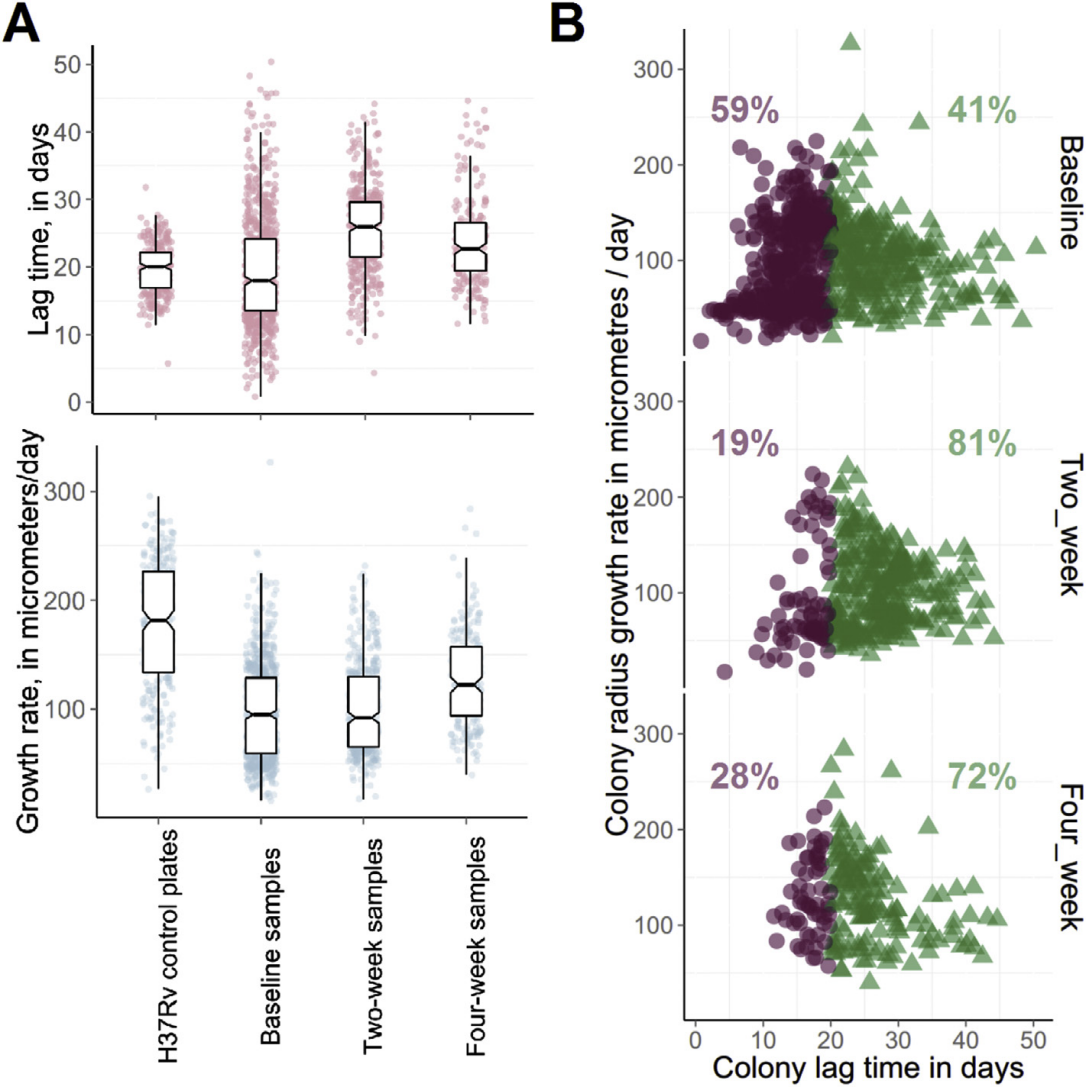
**Observed distributions of colonies' lag times and growth rates change over time on treatment**. A. Box plots showing lag times (top) and radial growth rates (bottom) of observed H37Rv plate colonies (n = 269), and observed colonies recovered from clinical isolates (n = 1352) at three time points into treatment (0, 2, and 4 weeks). Box shows median and interquartile range. B. Scatterplots of lag time and growth rate for colonies recovered from clinical isolates at the 3 treatment time points. Partitioning the colonies above and below the overall median lag time of 20 days, indicated by red circle and green triangle markers, shows that bacilli forming colonies with shorter lag time are the dominant sub-population at baseline, but are a minority after 2 or 4 weeks of treatment.

Distributions of observed colony lag times and growth rates are shown in Figure 1. Variation in colony lag times was notably greater for the clinical isolates compared to H37Rv control plates. For the clinical isolates, observed colonies' mean lag time was 19.4 days in baseline samples, 25.5 days in two-week and 23.8 days in four-week samples. An increase in mean colony lag time between baseline and two-weeks was seen for 8 out of 9 patients with colony growth captured at both these time points. Linear mixed effects modelling with a random ‘patient effect’ included suggested a fixed effect of lag time increasing by 6.5 days between baseline and two week colonies (95%CI 4.9–8.1 days, p = 0.0001, Figure 1A). Similarly, 5 out of 6 patients with available data showed an increase in mean colony lag time between baseline and four week samples; and linear mixed effects modelling estimated a fixed effect of lag time increasing by 4.7 days between baseline and four-week colonies (95%CI 3.6–5.7 days, p = 0.0267, Figure 1A).

There was no consistent pattern for mean RGR of observed colonies over time. Between baseline and week two samples, mean colony growth rate increased for 5 patients and decreased for 4.

When colonies were phenotyped by both lag time and RGR simultaneously, as in a scatter plot (Figure 1B), elimination over time remained selective, with the marked disappearance of short lag time colonies.

### 3.3 Corrected colony counts show different bacterial elimination dynamics for long versus short lag time colonies

**Figure 2:**
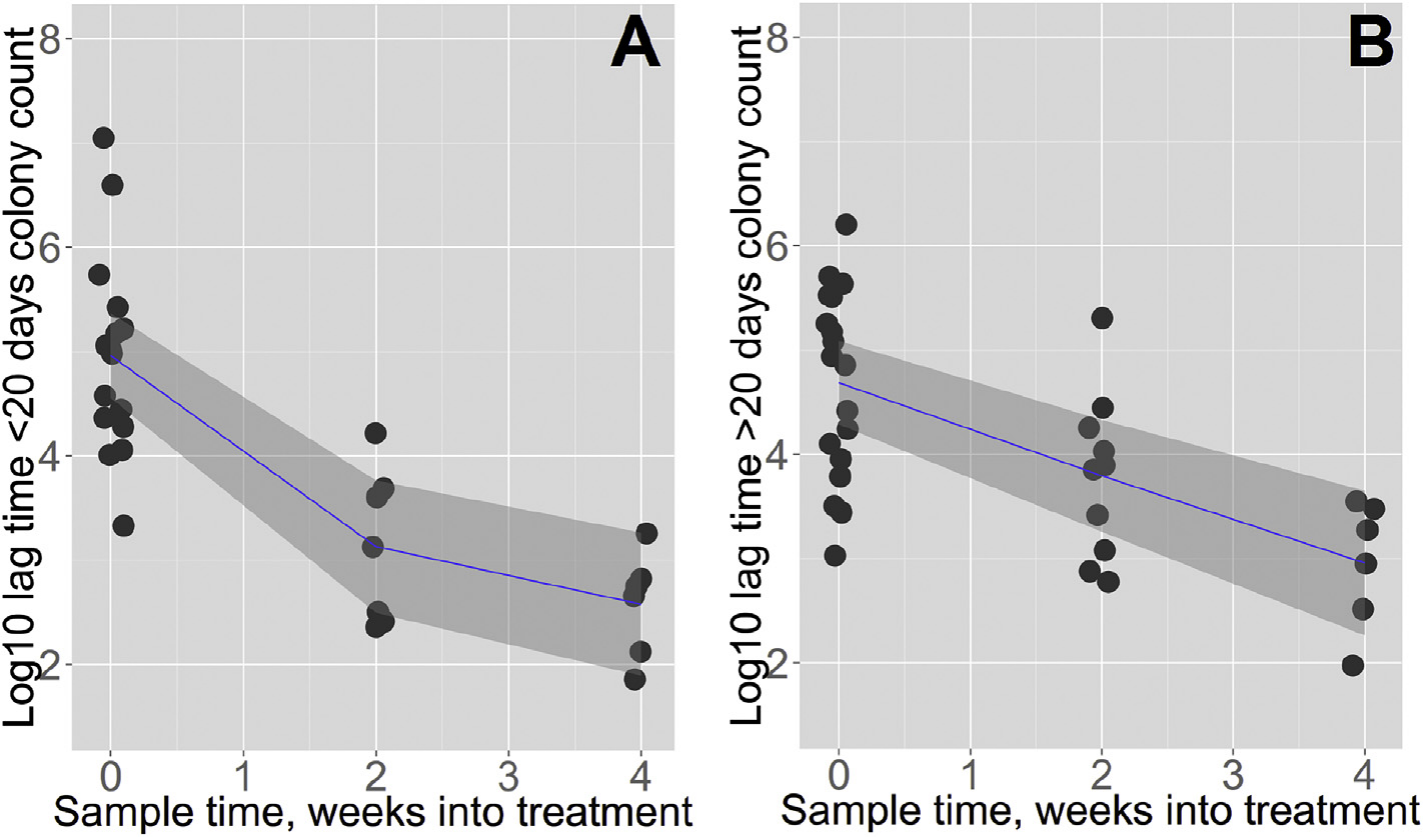
**CFU elimination from sputum over first 4 weeks treatment shows different dynamics for short and long lag time colonies.** Each data point represents a colony count for an individual patient sputum sample, averaged across usable plate replicates; each patient therefore contributes one colony count at each timepoint, less data points at later timepoints results from higher rates of culture negativity, contamination, and loss to follow up (n = 19 at 0 weeks, n = 10 at 2 weeks, n = 6 at 4 weeks). A. CFU count per ml sputum for patients at baseline, 2 weeks, and 4 weeks into treatment were multiplied by the proportion of observed colonies which had lag time ≤20 days in the sample, giving a ‘short lag CFU’ count per ml. A segmented line of best fit – allowed to bend at one point as shown – reduces residual sum of squares (RSS) compared to a single gradient line of best fit with a trend towards statistical significance for the improved fit (segmented linear regression RSS = 18.1, unsegmented linear regression RSS = 20.2; comparison by ANOVA, p = 0.09). B. CFU count per ml sputum multiplied by proportion of observed colonies with lag time >20 days in sample, giving elimination kinetics of ‘prolonged lag CFU’. Fitting a segmented line of best fit does not improve residual sum of squares compared with single gradient line of best fit (segmented linear regression RSS = 21.5, unsegmented linear regression RSS = 21.5; comparison by ANOVA, p = 0.93). Samples with zero short lag CFU (below limit of detection) were excluded to allow plotting on the log scale (n = 4 samples). Shaded areas show 95% confidence intervals around line of best fit.

Observed colony numbers were corrected for inoculum volume and dilution factor to give CFU count per ml sputum. The median total bacterial load fell by 1.5 log_10_ CFU/ml between baseline and week two, and by 0.5 log_10_CFU/ml between week two and four, consistent with a typical biphasic elimination. However, a different elimination dynamic was seen for sub-populations of CFUs categorised by colony lag time as ‘short-lag’ (≤20 days) or ‘long-lag’ (>20 days). Median count for short-lag colonies was 1.1 × 10^5^ CFU/ml at baseline, falling by 2.5 log_10_ to 300 CFU/ml by week 2. Over the same time period, median count for long-lag colonies fell by less than 1 log_10_ CFU/ml, from 7.2 × 10^4^ CFU/ml at baseline to 7500 CFU/ml at two weeks. Short-lag colonies therefore accounted for the steep decline in CFU/ml in the rapid, early bactericidal phase of treatment, while long-lag colonies showed a constant rate of elimination over the first 4 weeks of therapy (Figure 2).

### 3.4 TTD in MIGIT correlates with minimum colony lag time and CFU count

TTD in MGIT of each sputum sample with confirmed *M. tuberculosis* growth (n = 29) was compared to summary measures of solid media colony growth. As expected, samples with short TTD in MGIT had higher CFU count, with strong negative linear correlation between TTD and log_10_ CFU/ml (R^2^ 0.41, p = 0.0002). Minimum lag time for a discrete colony also correlated with TTD in liquid culture (R^2^ 0.18, p = 0.02). By contrast, mean lag time, minimum RGR, maximum RGR, and mean RGR all showed no correlation with TTD.

Minimum lag time on solid media and final colony count were strongly collinear, so their independent effects on TTD could not be reliably assessed in a multivariate analysis.

## 4 Discussion

This study is the first to quantitatively describe individual *M. tuberculosis* colony growth from clinical samples, and reports novel findings which suggest persister cells form colonies with longer lag times compared to bacilli which are rapidly eliminated by anti-tuberculosis treatment.

Pharmacodynamic modelling studies have previously reported biphasic elimination of sputum CFU counts over time on first-line TB treatment [23]. This may represent differential killing of bacillary sub-populations with metabolically quiescent organisms exhibiting antibiotic tolerance and surviving longer, and it has been proposed that modelling the slower second phase of elimination is a potential surrogate for long-term clinical outcome [11], [24]. The current study is the first to measure phenotypic heterogeneity of *M. tuberculosis* colonies obtained from sputum samples, and advances the sub-populations hypothesis by showing that distributions of colony growth kinetics vary non-randomly over the three time points assessed (baseline, week 2, week 4). In particular, counts of longer lag-time colonies (>20 days) decline more slowly than shorter lag-time colonies, and show linear decline when counts are aggregated across samples from different individuals.

One interpretation of these data is that a sub-population of *M. tuberculosis* cells infecting the human host are stochastically generated with long lag-time and relative drug tolerance before antibiotic insult, and become the dominant phenotype through population restructuring after antibiotic exposure. Such a bet-hedging strategy is described for other bacteria, and has been designated type II persister formation [25], [26]. For example, when bacteria are grown *in vitro* before inoculation onto solid media the distribution of resulting colony lag-times reflects the conditions of the original culture (e.g. nutrient restriction), and longer average lag times are associated with drug tolerance. By measuring individual colony growth for *E. coli* strains, Levin-Reisman et al. correlated the magnitude of the resulting long lag-time ‘tail’ with the proportion of bacteria able to survive antibiotic exposure [8]. Should the long lag-time colonies described in our analyses truly represent a metabolically quiescent sub-population of drug tolerant *M. tuberculosis* cells, their existence might directly reveal the mechanism of biphasic bacillary clearance, and measuring their elimination rate may help predict the risk of eventual TB treatment failure or post-treatment relapse.

Alternative explanations of our findings are possible. Long lag-time colony count dynamics may not reflect population restructuring, but simply that bacilli damaged by antibiotics have longer lag times, or a post-antibiotic effect is being observed. However, prior reports that treatment outcomes can be predicted from 2 month sputum culture conversion rate [27], or the rate of bacillary elimination from sputum [11], support the concept that type II persisters exist in sputum and that their phenotypic characteristics are likely to be relevant to clinical endpoints.

Serial sputum CFU counting is labour intensive, prone to experimental error, and shows systematic variation between laboratories. TTD in commercially available liquid culture systems is a convenient, readily standardised technique displaying moderate-strong correlation with colony counts, and is increasingly used as an alternative measurement. In addition, liquid culture has better sensitivity for reviving *M tuberculosis* at later timepoints in TB treatment, suggesting that it may be preferential for studying bacterial persistence [12], [28], [29]. However, TTD is a summary measurement – in which the fluorescent signal conferring a positive result is driven by oxygen consumption by the entire batch culture – and no prior studies have assessed its relationship with the growth kinetics of individual colonies. Our description of a correlation between TTD and minimum colony lag time on solid media raises the possibility that TTD is disproportionately influenced by the shortest-lag-time CFUs in clinical samples. Insofar as long lag-time colonies are an important contributor to bacterial persistence, overreliance on TTD may be unsatisfactory and it is important that serial CFU counting techniques are retained. Furthermore, the known non-linearity between log_10_ CFU count and TTD outside of the midrange [12], [13] could be partly explained by the confounding effect of CFU lag time distribution on the relationship between CFU count and TTD.

Despite the novel insights from our work, several limitations should be highlighted. Whilst our assessment of individual colonies generated a uniquely detailed dataset, the methods involved were labour-intensive and the number of patients included in the project was small. For the measure ‘proportion of colonies with lag time >20 days’, the unit of analysis is an individual sputum sample and only six patients had colony growth recorded from all three time points. In addition, the data is imbalanced, with some sputum samples' generating >10 fold more observed colonies than others. Larger studies, with longer follow-up will be required to confirm our exploratory findings and directly relate individual colony growth characteristics to clinical treatment response.

Overall, we have shown that phenotypic heterogeneity in *M. tuberculosis* colonies recovered from clinical specimens can be quantified using image analysis, providing novel pharmacodynamic insights. Slower elimination of CFU forming long lag time colonies suggests that these may represent type II persister bacilli. Further development of colony image analysis could prove useful in addressing fundamental research questions in tuberculosis drug therapy.

## Figures and Tables

**Table 1:** Summary of samples and colony growth by time of sample collection.

	Baseline (Week 0)	Week 2	Week 4	H37Rv colonies
**Number of patients**	21	13	12	–
**Solid media results:**				
Positive (CFU counted)	19	10	6	–
Negative or contaminated	2	3	6	–
**Liquid culture results:**				
Positive	17	7	5	–
Negative	2	3	1	–
Contaminated	2	3	6	–
**Summary of colonies' growth kinetics:**				
Lag-time, median (IQR)	17.9 (13.6–24.1)	25.9 (21.5–29.6)	22.7 (19.4–26.5)	20.0 (17.0–22.2)
Minimum lag time	2.1	4.3	11.6	5.7
RGR, median (IQR)	95 (59–129)	92 (65–130)	122 (93–157)	182 (134–226)
**Liquid culture TTD** median (IQR)	117 (107–145)	210 (147–308)	270 (267–304)	–

CFU = colony forming unit; IQR = interquartile range; RGR = radial growth rate of colony during initial linear growth phase, in micrometres per day; lag time = time in days from plate inoculation to initial colony growth, defined by x intercept extrapolated from initial observed colony growth; TTD = time to detection by MGIT broth culture, in hours.
