# Supplementary material for: Serial image analysis of Mycobacterium tuberculosis colony growth reveals a persistent subpopulation in sputum during treatment of pulmonary TB
Source: Tuberculosis (Edinb). 2016 May;98:110–5. doi: 10.1016/j.tube.2016.03.001 (PMC4869592; doi:10.1016/j.tube.2016.03.001)
Supplement: Supplementary file 1 [file mmc1.docx]

Online Supplement

**Figure S1. Colony growth digital imaging and analysis.**

1. Digital image of colony growth on solid media plate captured in custom made light box. Reference markers are seen which are used to ensure standardised placement of the dish in serial images.
2. Same image in OpenCFU software interface. Identified circular objects are highlighted in coloured boxes, and can be manually selected (blue squares) or deselected (red squares) for inclusion in final exported .csv file along with their measurements.


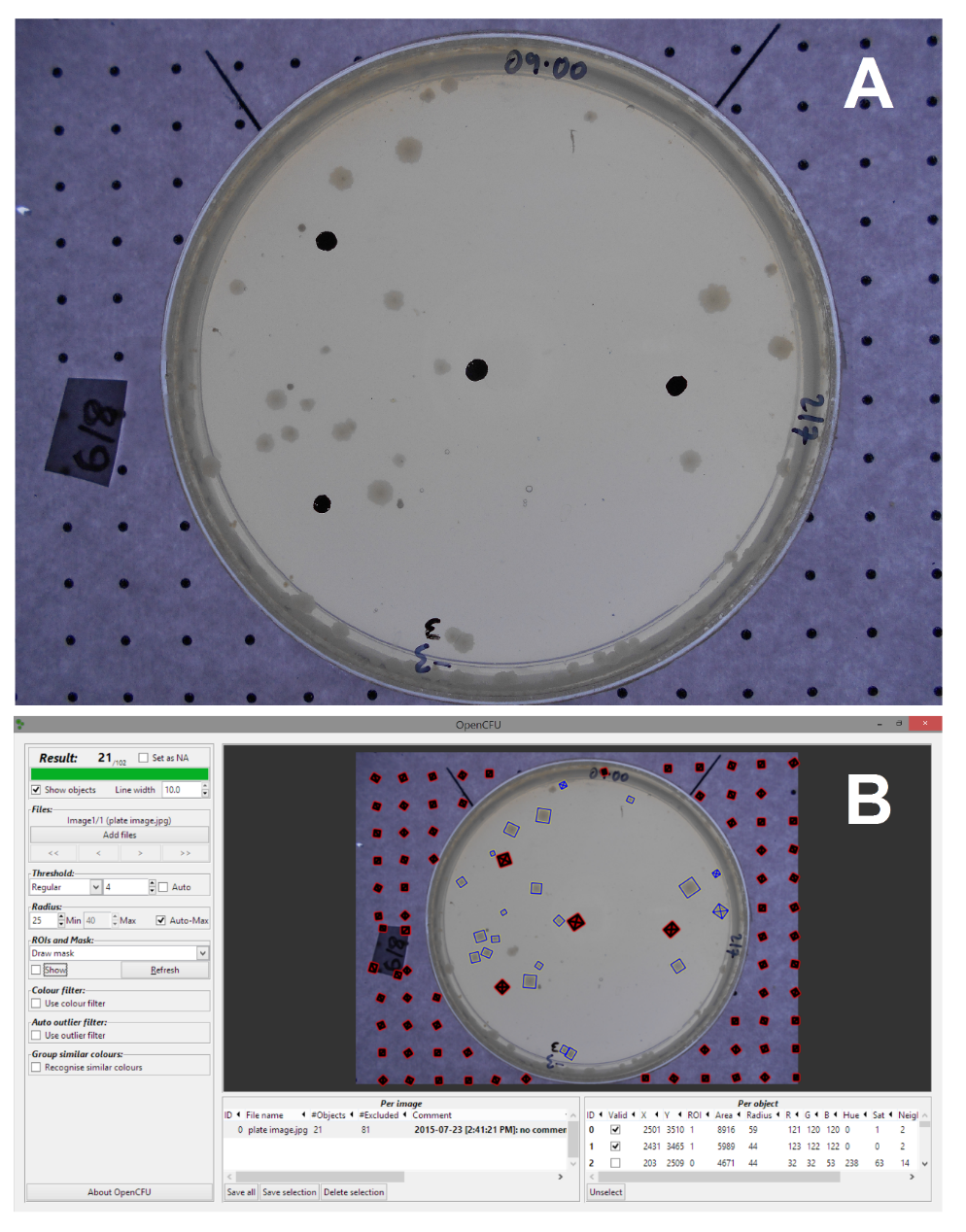


**Figure S2. Processing colony measurements to extract lag time and radial growth rate – schematic diagram.**

Serial measures of identified colonies, including radius and coordinates on image, are exported from OpenCFU in .csv file (A). Using an R programming script, measures of the same colony are identified in the .csv file from having similar x,y coordinates, and given a unique colony ID number (B). Individual colony growth curves are processed to extract initial linear growth rate and extrapolated x intercept using lmList function of nlme R package (C).

­­­


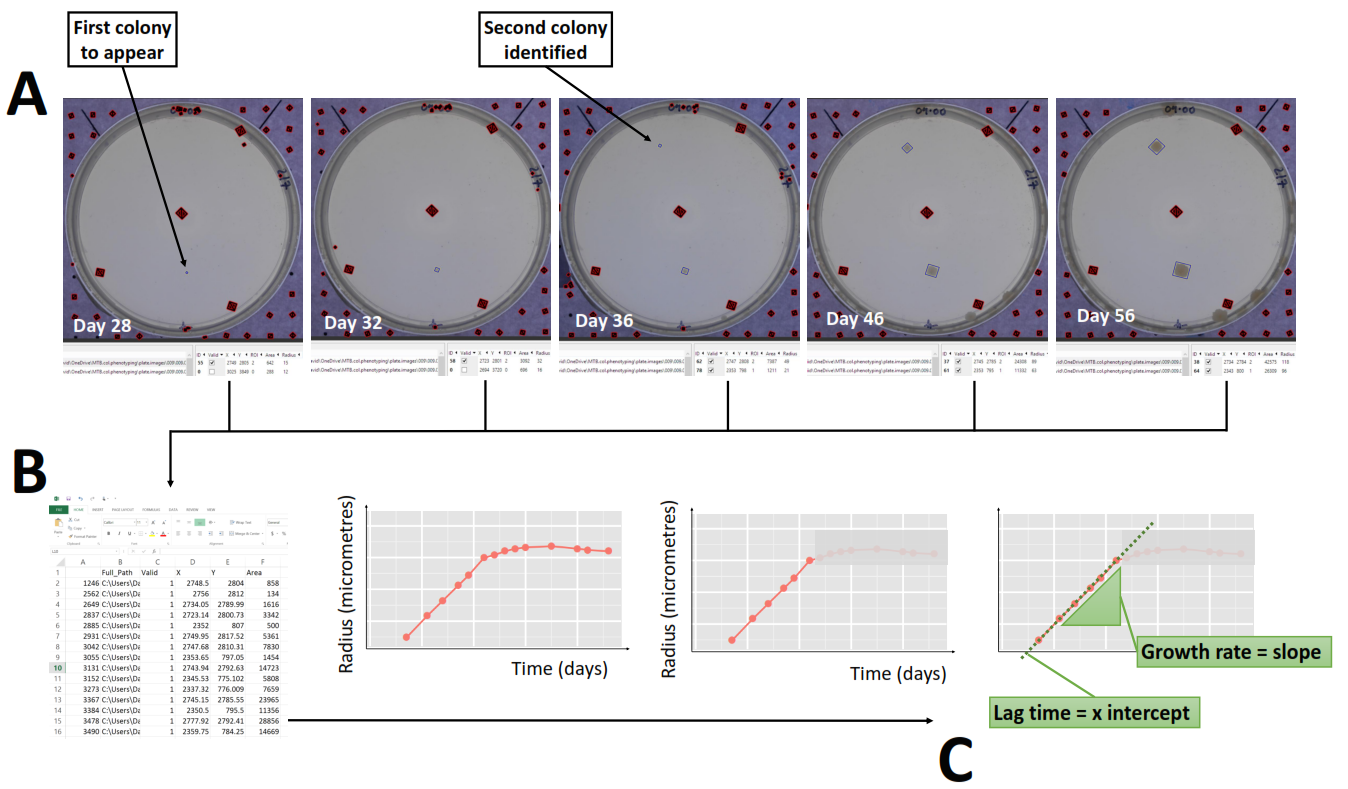


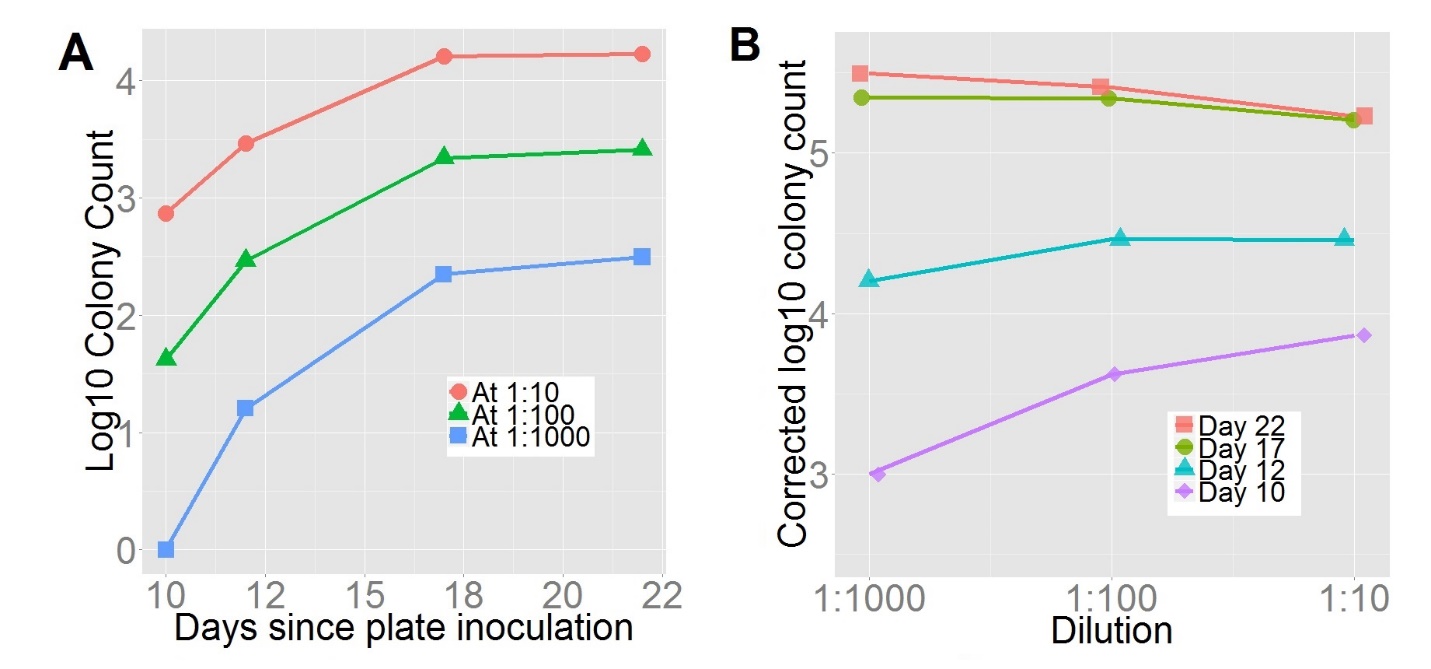


**Figure S3.** **Total colony counts for H37Rv control plates (n=45) by inoculum dilution (1:10, 1:100, 1:1000) and incubation time (days since plating).**

To investigate the effect of plating density (CFU concentration of the inoculate) on colony growth dynamics, a stock preparation of H37Rv *M. tuberculosis* lab strain was plated at three 10 fold dilutions, 1:10, 1:100, 1:1000. Total colony counts, summed across 15 replicate plates at each dilution, were calculated at 4 time points (10, 12, 17 and 22 days after inoculation).

**A**. At each dilution, number of colonies increases rapidly between day 10 and 18 as new colonies appear, and starts to plateau by day 22. At day 10 the difference in number of observed colonies between the 10 fold dilutions is greater than 1 log10, but at later time points the difference between the 10 fold dilutions is less than 1 log10.

**B**. The trivial part of the relationship between colony count and inoculum dilution is removed by correcting observed colony count for the dilution factor (1:10 plate counts multiplied by 10, 1:100 plate counts multiplied by 100, 1:1000 plate counts multiplied by 1000). At any given time point (represented by line colour) the corrected colony count should be the same at each dilution, giving a horizontal line. However, at day 10 the corrected colony count is higher in the least dilute plates (1:10) while by day 22 the most dilute plates (1:1000) have the highest corrected colony counts. Increasing the dilution of the inoculum increases colony lag time, but results in more colonies per unit of the original sample.

These observations show that plating density has an effect on colony growth dynamics: higher concentration of CFU is associated with a shortened lag time (consistent with quorum sensing behavior), but, after correction for dilution factor, ultimately form relatively fewer colonies (perhaps due to resource depletion, such as space on plate / available nutritients, or inhibition by metabolic products of established colonies). Such behavior has previously been described for mixed populations of soil microbes.[19]
